# Modulating the ERK1/2–MMP1 Axis through Corosolic Acid Inhibits Metastasis of Human Oral Squamous Cell Carcinoma Cells

**DOI:** 10.3390/ijms22168641

**Published:** 2021-08-11

**Authors:** Jen-Liang Chen, Chung-Yu Lai, Tsung-Ho Ying, Chiao-Wen Lin, Pei-Han Wang, Fang-Jung Yu, Chung-Jung Liu, Yi-Hsien Hsieh

**Affiliations:** 1 Department of Hematology & Oncology, Chung-Kang Branch, Cheng Ching Hospital, Taichung 40764, Taiwan; jenliangchen@yahoo.com.tw; 2 Director of Surgery Department, Chung-Kang Branch, Cheng Ching General Hospital, Taichung 40764, Taiwan; 6976@ccgh.com.tw; 3 Department of Obstetrics and Gynecology, Chung Shan Medical University Hospital, Taichung 40201, Taiwan; ying.steve@gmail.com; 4 Department of Obstetrics and Gynecology, School of Medicine, College of Medicine, Chung Shan Medical University, Taichung 40201, Taiwan; 5 Institute of Oral Sciences, Chung Shan Medical University, Taichung 40201, Taiwan; cwlin@csmu.edu.tw; 6 Institute of Medicine, Chung Shan Medical University, Taichung 40201, Taiwan; u9807410@gmail.com; 7 Division of Gastroenterology, Department of Internal Medicine, Kaohsiung Medical University Hospital, Kaohsiung Medical University, Kaohsiung 80708, Taiwan; yufj@kmu.edu.tw; 8 Department of Medicine, Faculty of Medicine, College of Medicine, Kaohsiung Medical University, Kaohsiung 80708, Taiwan; 9 Regenerative Medicine and Cell Therapy Research Center, Kaohsiung Medical University, Kaohsiung 80708, Taiwan; 10 Department of Medical Research, Chung Shan Medical University Hospital, Taichung 40201, Taiwan

**Keywords:** corosolic acid, human oral squamous cell carcinoma cells, metastasis, ERK1/2, MMP1

## Abstract

Corosolic acid (CA; 2α-hydroxyursolic acid) is a natural pentacyclic triterpenoid with antioxidant, antitumour and antimetastatic activities against various tumour cells during tumourigenesis. However, CA’s antitumour effect and functional roles on human oral squamous cell carcinoma (OSCC) cells are utterly unknown. In this study, our results demonstrated that CA significantly exerted an inhibitory effect on matrix metalloproteinase (MMP)1 expression, cell migration and invasion without influencing cell growth or the cell cycle of human OSCC cells. The critical role of MMP1 was confirmed using the GEPIA database and showed that patients have a high expression of MMP1 and have a shorter overall survival rate, confirmed on the Kaplan–Meier curve assay. In the synergistic inhibitory analysis, CA and siMMP1 co-treatment showed a synergically inhibitory influence on MMP1 expression and invasion of human OSCC cells. The ERK1/2 pathway plays an essential role in mediating tumour progression. We found that CA significantly inhibits the phosphorylation of ERK1/2 dose-dependently. The ERK1/2 pathway played an essential role in the CA-mediated downregulation of MMP1 expression and in invasive motility in human OSCC cells. These findings first demonstrated the inhibitory effects of CA on OSCC cells’ progression through inhibition of the ERK1/2–MMP1 axis. Therefore, CA might represent a novel strategy for treating OSCC.

## 1. Introduction

Oral squamous cell carcinoma (OSCC) is the most common malignant subgroup of head and neck cancers. OSCC is life threatening, mainly occurs between 40 and 70 years of age and is the leading cause of death, particularly in men. OSCC, which includes cancers of the lips, tongue, cheeks, gingiva, the floor of the mouth, buccal mucosa, palate and pharynx, is associated with risk factors including alcohol consumption, betel quid chewing, radiation and viral infections [1,2]. The invasion and metastasis of OSCC cells are factors in the high mortality rate [3,4]. Thus far, invasion and metastasis are still the target issues for improving the poor clinical outcomes and high mortality rates in patients with cancer. Metastasis accelerates cancer progression through multiple events involving cell migration, cell invasion, angiogenesis and extracellular matrix (ECM) disruption [5,6]. ECM degradation by extracellular proteinases is crucial for tumour cell invasion, metastasis and malignant progression [7]. Thus, the prevention/inhibition of ECM degradation through targeting proteinases, such as matrix metalloproteinases (MMPs), is considered a critical step for cancer therapy [8,9].

MMPs are a family of zinc-dependent endopeptidases and are overexpressed in several malignant tumours. Among MMPs, MMP1 is known as collagenase and degrades collagen type I and III by recognizing the substrate through a hemopexin-like domain [10]. MMP1 is highly expressed in driving tumour progression in aggressive lung cancer [11] and contributes to the migration and invasion of hepatocellular carcinoma cells [12]. MMP1 is also upregulated to promote an invasive brain phenotype in metastatic breast cancer cells [13]. Therefore, targeting MMP1 is considered an essential step for suppressing cancer progression. The effects of corosolic acid (CA) on MMP1 expression and the invasive motility of OSCC cells, the related molecular mechanisms behind CA-antitumour, are investigated in detail in this study.

Potential natural phytochemicals against cancers have been investigated due to the properties of less toxicity and more efficient treatment. CA, a natural pentacyclic triterpenoid, is the principal component of *Lagerstroemia speciosa* leaves (also called Banaba). Accumulating studies have indicated that CA exerts biological properties including anticancer, antidiabetes, antiobesity, antiinflammation, antihyperlipidaemic and antiviral effects [14]. CA induces ER-stress-dependent apoptosis in human prostate cancer cells [15]. In addition, CA inhibits cancer progression through inactivating the Yes-associated protein (YAP) in hepatocellular carcinoma [16]. However, CA’s antitumour effects and molecular mechanisms in human OSCC cells have not yet been investigated. We determined that corosolic acid (CA) has antitumor effects against human OSCC cells, including growth inhibition, cell cycle arrest, apoptosis induction, migration and invasion suppression. Next, we identified the molecular mechanism behind CA-inhibited OSCC progression. This study demonstrated that CA inhibits cell migration and invasion by inhibition of the ERK1/2–MMP1 axis in human OSCC cells.

## 2. Results

### 2.1. Effect of CA on Cell Viability and Colony Formation of OSCC Cells

**Figure 1:**
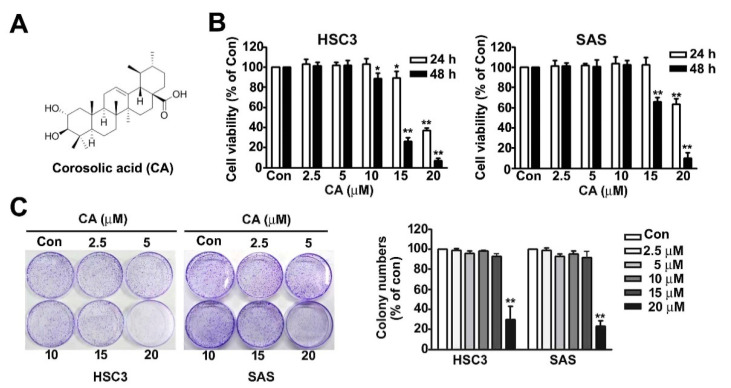
Effect of CA on the viability and cytotoxicity of human HSC3 and SAS OSCC cells. (**A**) Structure of corosolic acid (CA). (**B**) Human HSC3 and SAS cells were exposed to various concentrations (0, 2.5, 5, 10, 15 and 20 μM) of CA for 24 and 48 h and then measured to observe cell viability using MTT assay. (**C**) The cell proliferation rates of human HSC3 and SAS cells exposed with CA (0, 2.5, 5, 10, 15 and 20 μM) for 5 days were measured using a colony formation assay. *, *p* < 0.05; **, *p* < 0.01 vs. control (line 1), (Mean ± SE, *n* = 3). Control: untreated cells.

The CA (2α-hydroxyursolic acid) structure is shown in Figure 1A. The influence of CA on cell viability and colony formation was studied in two cell lines, HSC3 (human tongue squamous carcinoma cell line) and SAS (human tongue squamous carcinoma cell line). Both HSC3 and SAS cell lines were treated with various concentrations (0, 2.5, 5, 10, 15 and 20 μM) of CA for 24 or 48 h, after which cellular viability was analysed using the 3-(4,5-dimethylthiazol-2-yl)-2,5-diphenyl-tetrazolium bromide (MTT) assay. Cell viability was significantly decreased using CA (15 and 20 μM) in the HSC3 and SAS cell lines at 24 and 48 h (Figure 1B) but did not affect cell growth in DMSO (0.04%)-treated HSC3 and SAS cells (Supplementary Figure S1A). The cell proliferation rate was further measured using a colony formation assay to confirm the inhibitory effect on the growth of HSC3 and SAS cells exposed to CA (0, 2.5, 5, 10, 15 and 20 μM) for 5 d (Figure 1C). These findings revealed that high concentrations of CA (15 and 20 μM) exerted a cytotoxic effect on the growth of human OSCC cells. Therefore, we used CA at concentrations <15 μM in the subsequent experiments.

### 2.2. Effect of CA on Cell Cycle Distribution and Apoptosis Induction in Human HSC3 and SAS Cells

**Figure 2:**
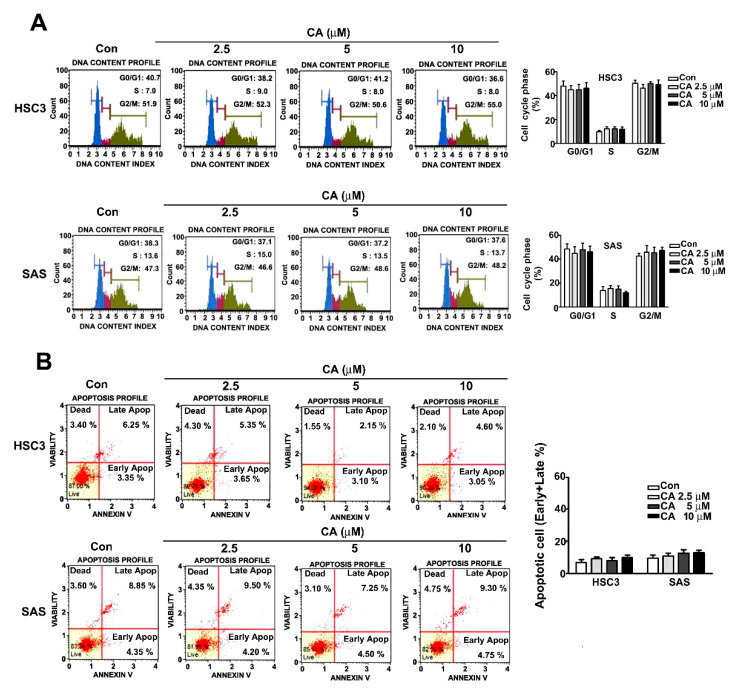
Effect of CA on cell cycle arrest and apoptosis induction in human HSC3 and SAS OSCC cells. (**A**) The regulation of cell cycle distribution was analysed using PI staining by flow cytometry. (**B**) Apoptosis induction in human HSC3 and SAS cells exposed to various concentrations (0, 2.5, 5 and 10 μM) of CA by Annexin V/PI staining through flow cytometry. (Mean ± SE, *n* = 3). Control: untreated cells.

Human HSC3 and SAS cells were exposed to various concentrations (0, 2.5, 5 and 10 μM) of CA for 24 h. Cell cycle distribution and apoptosis induction were further analysed using a flow cytometry assay. The results revealed that CA treatment (0, 2.5, 5 and 10 μM) did not influence the regulation of cell arrest at any phase (Figure 2A). Moreover, the induction of apoptosis using CA was not observed in HSC3 and SAS cells through flow cytometry detection (Figure 2B). Therefore, CA does not affect the cell cycle distribution and induction of apoptosis in human OSCC cells based on the aforementioned results.

### 2.3. CA Suppresses Cell Migration and Invasion in Human OSCC Cells

**Figure 3:**
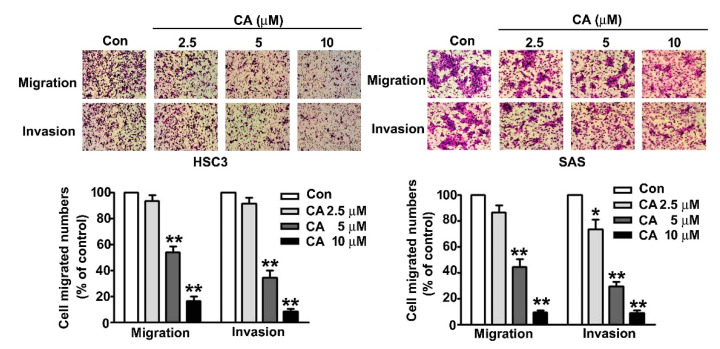
Effect of CA on cell migration and invasion of human HSC3 and SAS OSCC cells. Human HSC3 and SAS cells were treated with various concentrations of CA (0, 2.5, 5 and 10 μM) for 24 h, and then, capacities of cellular migration and invasion were measured. *, *p* < 0.05, **, *p* < 0.01 vs. control (line 1), (Mean ± SE, *n* = 3). Control: untreated cells. Scale bars: 50 μm.

To investigate the influence of CA in regulating cellular migration and invasion activity in human OSCC cells, human HSC3 and SAS cells were exposed to various concentrations of CA (0, 2.5, 5 and 10 μM) for 24 h and subjected to cellular migration and invasion assays. The findings showed that CA significantly suppresses the cell migration and invasion activity of human HSC3 and SAS cells dose-dependently (Figure 3).

### 2.4. CA Inhibits MMP1 Expression and Cell Invasion in Human HSC3 and SAS OSCC Cells

**Figure 4:**
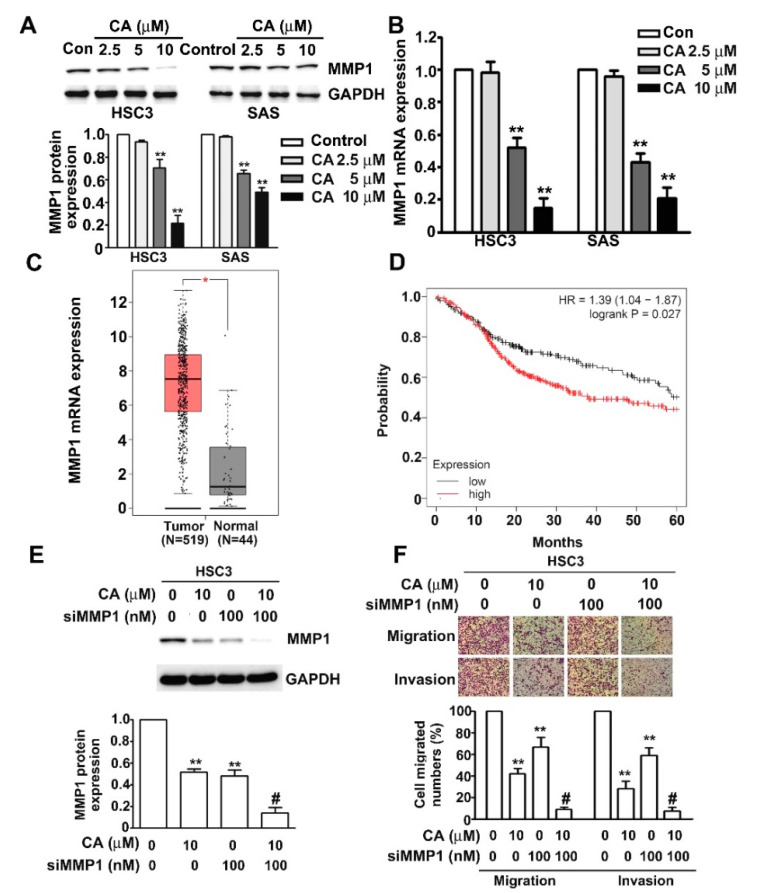
Effect of CA on MMP1 expression and cell invasion in human HSC3 and SAS OSCC cells. (**A**,**B**) Human HSC3 and SAS cells were treated with various concentrations of CA (0, 2.5, 5, 10 or 20 μM) for 24 h, after which the protein and mRNA expression of MMP1 was measured via immunoblotting and qRT-PCR analysis. (**C**) MMP1 mRNA expression in the matched OSCC tissues and normal tissues was validated from the GEPIA databases. T: Tumour tissue (*n* = 519); N: Normal tissue (*n* = 44). *, *p* < 0.05 versus normal tissue. (**D**) The red line indicates high MMP1 expression, and the black line indicates low MMP1 expression. (**E**,**F**) Human HSC3 cells were treated with CA (10 μM), siMMP1 (100 nM) or CA (10 μM) plus siMMP1 (100 nM); then, MMP1 protein expression, migration and invasion were measured. GAPDH as a protein-loading control. **, *p* < 0.01 versus control (line 1), #, *p* < 0.05 vs. CA (line 2) or siMMP1 (line 3). Control: untreated cells. Scale bars: 50 μm.

MMP1 has been reported to act as a critical factor in accelerating cancer progression by upregulating the capacity of migration and invasion in cancer cells [12]. We treated both human HSC3 and SAS cells with various concentrations of CA (0, 2.5, 5, 10 or 20 μM) for 24 h and identified the influence of CA on the protein and mRNA expressions of MMP1 in these cells using immunoblotting and RT-qPCR assays. The results revealed that CA significantly reduced the protein and mRNA levels of MMP1 in HSC3 and SAS cells (Figure 4A,B). Higher MMP1 mRNA expression of human OSCC tissues than that of normal oral tissues was further confirmed using the GEPIA database (Figure 4C). Furthermore, patients with OSCC with high MMP1 expression had shorter overall survival (OS) (HR = 1.39, 95% CI 1.04–1.87, *p* = 0.027) than those with low MMP1 expression (Figure 4D). We further examined the synergistic influence of both CA and siMMP1 on the MMP1 expression, and migration and invasion activity in human HSC3 and SAS cells. The results revealed that an individual treatment of CA (10 μM) or MMP1 siRNA (siMMP1; 100 nM) significantly downregulated the MMP1 protein expression, and migration and invasion activity in human HSC3 cells. The scramble siRNA did not affect the MMP1 expression (Supplementary Figure S2A). Additionally, the co-administration of CA (10 μM) and siMMP1 (100 nM) showed a greater synergistic inhibitory effect on MMP1 expression and motility activity in human HSC3 cells (Figure 4E,F).Transfection of the scramble siRNA did not affect the migration and invasion ability of HSC3 cells (Supplementary Figure S2B).

### 2.5. ERK1/2 Pathway Mediates CA-Downregulated MMP1 Expression, Cell Migration and Invasion in Human HSC3 Cells

**Figure 5:**
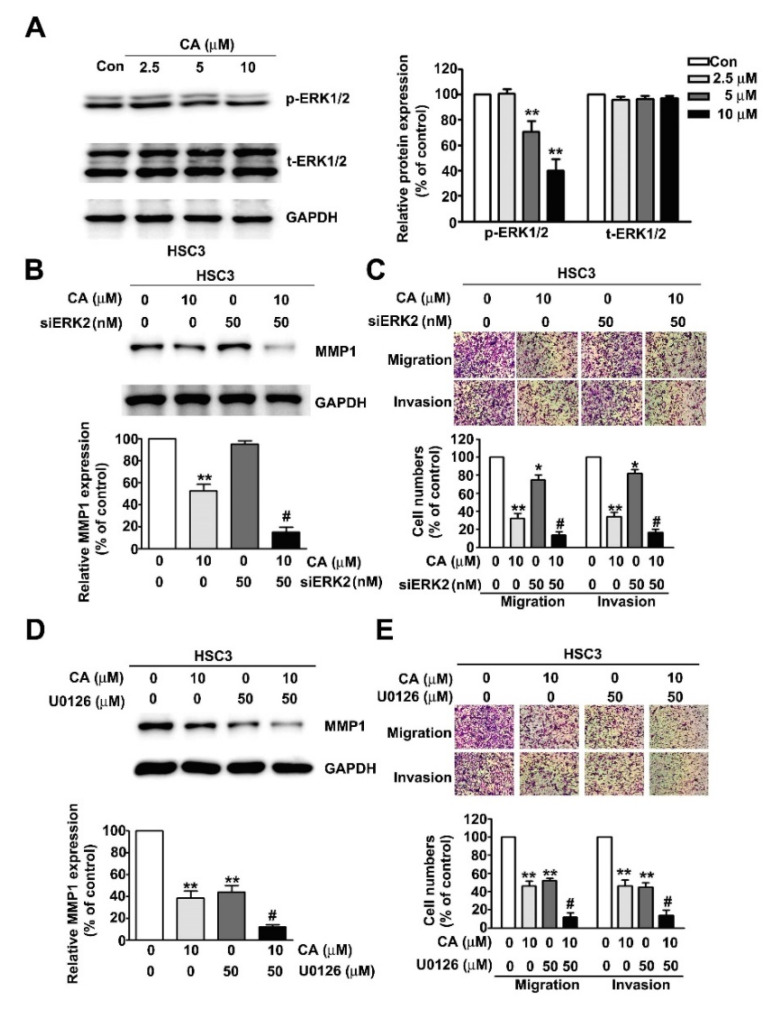
ERK1/2 pathway participation in CA-downregulated MMP1 expression, cell migration and invasion in human HSC3 cells. (**A**) Human HSC3 cells were treated with various concentrations of CA (0, 2.5, 5 or 10 μM), after which phospho-ERK1/2 and total-ERK1/2 protein levels were measured via immunoblotting analysis. (**B**,**C**) Human HSC3 cells were treated with CA (10 μM), siERK2 (50 nM) or CA (10 μM) plus siERK2 (50 nM); and then MMP1 protein level, migration and invasion were further measured. (**D**,**E**) Human HSC3 cells were treated with CA (10 μM), U0126 (specific ERK1/2 inhibitor; 50 μM) or CA (10 μM) plus U0126 (50 μM), following which MMP1 protein level, migration and invasion were further measured. GAPDH was used as a protein loading control. Control: untreated cells and scrambled siRNA-transfected cells (for siRNA transfection). *, *p* < 0.05; **, *p* < 0.01 versus control (line 1); #, *p* < 0.05 versus CA (line 2) or siERK2, U0126 (line 3) (Mean ± SE, *n* = 3). Control: untreated cells. Scale bars: 50 μm.

The ERK1/2 pathway plays an essential role in mediating tumour progression [12,17]. Thus, we examined the influence of CA on ERK1/2 activation of human HSC3 OSCC cells treated with various concentrations of CA (0, 2.5, 5 or 10 μM). The results revealed that CA significantly inhibits ERK1/2 phosphorylation dose-dependently (Figure 5A). ERK2 siRNA (siERK2) and U0126 (ERK1/2 specific inhibitor) were further used to confirm the ERK1/2-mediated inhibitory effect on MMP1 expression and invasive motility in human HSC3 cells. The synergistically inhibitory effects of CA (10 μM) plus siERK2 (50 μM) or CA (10 μM) plus U0126 (50 μM) co-treatment on MMP1 expression and metastatic activity were then measured in human HSC3 cells (Figure 5B–E). The scramble siRNA did not affect the ERK2 expression and cell migration/invasion in HSC3 cells (Supplementary Figure S2C,D).The results indicated that the ERK1/2 pathway plays a critical role in mediating CA-downregulated MMP1 expression and metastasis in human HSC3 cells.

## 3. Discussion

OSCC is currently the fourth leading cause of cancer-related deaths among men in Taiwan. OSCC is life threatening because the five-year survival rate of total OSCC patients is approximately 50% [18]. Therefore, it is crucial to identify the potential treatment strategies and anticancer drugs for OSCC prevention and therapy. Natural phytochemicals are worth developing because compounds of plant origin have less toxicity and a more potential biological activity against cancer progression. CA is a natural pentacyclic triterpenoid and can be extracted from the leaves of *Eriobotrta japonica* [19] and from the fruit of *Cratoegus pinnatifida* var. Psilosa [20]. In this study, the results were as follows: (1) CA did not reduce cell growth and colony formation by regulating cell cycle arrest or apoptosis induction in human OSCC cells; (2) CA significantly inhibits cell migration and invasion by downregulating MMP1 expression; (3) MMP1 is highly expressed in OSCC tissues and is positively correlated with shorter OS of patients with OSCC; (4) the co-administration of CA and siMMP1 synergistically inhibits MMP1 expression and the invasive motility activity of human OSCC cells; and (5) the ERK1/2 pathway mediates CA-downregulated MMP1 expression, cell migration and invasion in human OSCC cells. Collectively, these findings indicate that CA inhibits invasive motility by downregulating the ERK1/2–MMP1 axis. Therefore, CA could rather potentially be a novel and interesting natural phytochemical agent against OSCC.

The poor prognosis and high mortality of OSCC are primarily due to the highly invasive motility activity in cancer cells. ECM remodelling via MMP activation contributes to the spread and invasion of malignant cells by disrupting the interaction between cells and the ECM [10]. Immunohistochemistry with the use of a tissue microarray revealed that MMP1 is overexpressed in primary nodular melanoma [21]. MMP1 overexpression by silencing miR-202-3p promotes breast cancer cells to transmigrate through the brain endothelium [13]. The activation of COX2-MMP1 signalling via miR-101-3p loss promotes the transmigration of metastatic breast cancer cells through brain endothelium [22]. MMP1 drives tumour progression in large cell carcinoma of the lung through fibroblast senescence [11]. Thus, the inhibition of ECM remodelling by targeting MMP1 activation is considered a critical step in cancer therapy. Interferon regulatory factor 2 inhibits gastric cancer invasion and migration by downregulating MMP1 [23]. The knockdown of MMP1 inhibits the progression of colorectal cancer by suppressing the PI3K/Akt/c-myc signalling pathway and epithelial–mesenchymal transition [24]. miR-361-5p inhibits the glycolysis, proliferation, and invasion of breast cancer cells by targeting MMP1 [25]. The reduction in PKC alpha expression inhibits cell proliferation, migration and invasion in human malignant hepatocellular carcinoma (HCC) through downregulating MMP1 expression [26]. Licochalcone A inhibits the migration and invasion of human lung cancer cells via the downregulation of MMP1 expression [27]. Praeruptorin A inhibited the migration and invasion of human HCC cells while downregulating the expression of MMP1 [12]. The expression of MMP1 is upregulated in oral lichen planus, dysplasia, squamous cell carcinoma, and lymph node metastasis [28]. The relative mRNA level of MMP1 in the OSCC tissues has a 3.26-fold increase in comparison with that in paired normal tissues [29]. The elevated MMP1 protein expression is associated with a higher histopathological grade of OSCC [30]. Tight junction protein claudin-1 enhances the invasive activity of OSCC by promoting the cleavage of a laminin-5 gamma2 chain via MMP1 [31]. S100A14 regulates the invasive potential of OSCC-derived cell lines in vitro by modulating the expression of MMP1 [32]. MMP1 3′UTR accelerates the proliferation and migration of OSCC by sponging miR-188-5p to upregulate the expression of SOX4 and CDK4 [33]. This study observed that MMP1 is highly expressed in OSCC tissues compared with that in normal oral tissues. A high expression of MMP1 was positively correlated with a shorter OS rate of patients with OSCC. MMP1 downregulation using CA or CA cotreatment with siMMP1 significantly inhibits migration and invasion in human OSCC cells.

ERK1/2 is a member of mitogen-activated protein kinases and is reported to play a pivotal role in regulating tumorigenic processes in vitro and in vivo, including cellular proliferation, apoptosis, angiogenesis, lymphangiogenesis and metastasis. ERK1/2 is showed to mediate fisetin-induced apoptosis and anticancer effects in human cervical cancer HeLa cells [34]. The inhibition of ERK1/2 via U0126 or transfection with the siERK plasmid significantly abolishes the fisetin-inhibited migration and invasion by activating the ERK1/2 pathway [35]. ERK1/2 blocking inhibits MMP-2-mediated cell motility and further enhances the anti-invasive ability of tricetin in glioblastoma multiforme cells [36]. Praeruptorin A significantly inhibits the growth and invasion of human cervical cancer cells by suppressing ERK1/2 signalling [17]. Epigallocatechin gallate could inhibit the migration of human uveal melanoma cells via the downregulation of ERK1/2 phosphorylation [37]. Kaempferol inhibits cell migration by targeting ERK1/2 signalling in human retinal pigment epithelial cells [38]. In addition, ERK1/2 mediates the antimetastatic activity of β-mangostin against human hepatocellular carcinoma cells [39]. This study observed that the inhibition of ERK1/2 signalling via siERK2 or U0126 significantly suppressed MMP1 expression, migration and invasion in human OSCC cells. The co-administration of CA plus siERK2 or CA plus U0126 showed a greater synergistic inhibitory effect on MMP1 expression and cellular motility activity.

Potential plant-derived bioactive compounds with less toxicity and more therapeutic effects are identified as auxiliary anticancer therapy for improving the outcome of patients with cancer. CA decreases intracellular β-catenin levels and suppresses APC-mutated colon cancer cell growth [40]. Additionally, CA administration reduces the number of infiltrating lymphocytes in tumour tissues. A significant immunosuppressive effect of myeloid-derived suppressor cells in tumour-bearing mice was abrogated using CA in ex vivo analysis [41]. CA exhibits antiangiogenic and antilymphangiogenic effects by reducing the proliferation and migration of human umbilical vein endothelial cells stimulated using angiopoietin-1 on the in vitro model. CA decreases the final tumour volume and the blood and lymphatic vessel densities of tumours, indicating that it suppresses in vivo angiogenesis and lymphangiogenesis on an in vivo CT-26 colon carcinoma animal model [42]. CA inhibits colorectal cancer cell growth via directly targeting HER2 and HER3 heterodimerisation [43]. CA treatment reduces tumourigenesis through translocating and deactivating YAP from the nucleus in hepatocellular carcinoma [16]. CA inhibits VEGFR2 kinase activity, disrupts tubulin structure and impairs human lung adenocarcinoma A549 cell proliferation in a xenograft mouse model [44]. CA inhibits cell growth and induces ER stress-dependent apoptosis in human prostate cancer PC-3 and DU145 cell lines and decelerates tumour growth in a xenograft model [15]. CA treatment dose-dependently induces cytotoxicity, cell cycle arrest and apoptosis in human retinoblastoma Y-79 cells by disrupting MELK-FM1 signalling [45]. CA induces non-apoptotic cell death in other renal cancer cells (ACHN and A498), breast cancer cells (MDA-MB231) and hepatocellular carcinoma cells (SK-Hep1 and Huh7) through the generation of lipid reactive oxygen species production [46]. CA is reported to have a lowering effect on postprandial blood sugar levels in human studies and to improve insulin resistance conditions [47,48]. Some evidence has shown that no adverse effects about CA used in optimal dosage are reported in clinical trials and animal study. CA may resolve metabolic syndrome and may provide health benefits [47,49]. Thus, CA is considered as a potential compound against OSCC treatment.

## 4. Materials and Methods

### 4.1. Chemical Reagents and Antibodies

Corosolic acid (CA: formula: C30H48O4) was purchased from ChemFaces company (Wuhan, Hubei, China). MTT and DMSO were obtained from Sigma-Aldrich (St. Louis, MO, USA). Primary antibodies against phospho-EKR1/2, total-ERK1/2, MMP1 and GAPDH were purchased from Cell Signalling Technology (Danvers, MA, USA). The scramble siRNA and siRNA-MMP1 (siMMP1) were obtained from Santa Cruz Biotechnology (Santa Cruz, CA, USA). The scramble siRNA and siRNA-ERK2 (siERK2) was purchased from ALLBio (Taipei, Taiwan).

### 4.2. Cell Culture

Human HSC3 (Human tongue squamous carcinoma cell line) and SAS (Human tongue squamous carcinoma cell line) were a gift from Prof. Shun-Fa Yang (Institute of Medicine, Chung Shan Medical University, Taichung, Taiwan). HSC3 and SAS cells were cultured in DMEM/F-12 medium (Life Technologies, Grand Island, NY, USA) containing 10% foetal bovine serum, 2 mM glutamine and 100 U/mL of penicillin–streptomycin (Invitrogen Life Technologies, Carlsbad, CA, USA). All cell cultures were maintained in a humidified incubator with 5% CO_2_ at 37 °C. An origin stock reagent was prepared for CA (50 mM) and dissolved in dimethyl sulfoxide (DMSO) solution. The cell experiment used final DMSO concentrations for the highest concentration of CA at 0.04% DMSO.

### 4.3. Cell Viability Assay

To determine the effect of CA on cell viability, human OSCC cells were seeded with a density of 9 × 10^4^/well (HSC3) or 1.2 × 10^5^/well (SAS) cells per well in 24-well plates (Greiner Bio-One, Frickenhausen, Germany) and treated with various concentrations of CA for 24 or 48 h. The cells were then washed with PBS and incubated at a final concentration of 0.5 mg/mL of MTT per well at 37 °C in 5% CO_2_ for 4 h. Cell viability was measured at 563 nm using a Multiskan MS ELISA reader (Labsystems, Helsinki, Finland).

### 4.4. Colony Formation Assay

Colony formation was performed as described in previous studies [50]. Human HSC3 and SAS cells were seeded in six-well plates (1 × 10^4^/well) and treated with various concentrations of CA for 5 days. More than 100 colonies were stained with 0.5% crystal violet solution for 30 min at room temperature and analysed. Three independent experiments were performed.

### 4.5. Annexin V/PI Staining via Flow Cytometry Analysis

Cell cycle assay was performed as described in a previous study [51]. Human HSC3 and SAS cells (5 × 10^5^/well) were treated with various concentrations of CA for 24 h and then fixed with 75% ice ethanol overnight. These fixed cells were stained with PI reagent for 20 min. Cell DNA content was measured through flow cytometry using the Muse Cell Analyser (Merck Millipore, Burlington, MA, USA). The outcome data were further analysed using the Muse Cell Analyser. An apoptosis assay was performed as previously described [52]. After the HSC3 and SAS cells were treated with various concentrations of CA for 24 h, the cells were harvested and apoptosis was measured using the Muse Annexin V and Dead Cell Assay Kit (Merck Millipore). In brief, the collected cells were incubated with 5 μL of Annexin V-FITC and 5 μL of PI reagents at room temperature in darkness for 15 min. The apoptotic cell population was then analysed using the Muse Cell Analyser (Merck Millipore).

### 4.6. siRNA Transfection

The siRNA transfection assay were performed as previously as published report [12]. For the knockdown assay, 4 × 10^5^ of OSCC cells were seeded into a 6 cm culture dish overnight. Using the RNAiMAX reagent (Thermo Fisher Scientific, MA, USA), scramble siRNA or siRNA-MMP1 (siMMP1)/siRNA-ERK2 (siERK2) were mixed in serum free medium for 6 h. A fresh culture medium (20% FBS) was then added and incubated in a 37 °C. After 24 h, it was treated with or without CA (10 μM) for another 24 h and detected for subsequent experiments.

### 4.7. Immunoblotting Analysis

Immunoblotting analysis was performed as described previously [51]. Briefly, proteins were harvested from the human HSC3 and SAS cells lysed with a lysis buffer. Equal amounts of total protein (20 μg) from each experimental group were subjected to 10–12% SDS-PAGE for protein separation and then transferred onto a PVDF membrane (Life Technologies, Carlsbad, CA, USA). Next, the membranes were blocked with 5% non-fat dry milk in Tris-buffered saline with Tween-20 buffer. The blocked membranes were further incubated with primary target antibodies and subsequently with secondary antibodies to detect antibody-bound protein bands using the Luminescent Image Analyser (LAS 4000 mini, GE Healthcare Bio-Sciences, Pittsburgh, PA, USA).

### 4.8. Migration and Invasion Assay

Cellular migration and invasion were analysed as described previously [51]. In brief, HSC3 and SAS cells (5 × 10^5^/well) were seeded onto filter inserts (pore size: 8 μm) that were pre-coated with or without Matrigel (0.5 mg/mL) for cellular invasion assay and migration assays, respectively. Cells migrating or invading the lower side of the filter insert were stained with 5% Giemsa reagent and counted at 200× magnification. Four microscopic fields were counted for each filter, and each experimental group was repeated three times.

### 4.9. Clinical Data Analysis by TCGA Database

The expression data from human OSCC tissues and normal oral tissues from TCGA and the GTEx projects were obtained by using a Gene Expression Profiling Interactive Analysis (GEPIA) database (http://gepia.cancer-pku.cn/, accessed on 1 July 2021), which can analyse the MMP1 expression of tumour and normal tissues. The Overall survival (OS) plots module was applied to examine the survival rate with high or low expression of MMP1 in human OSCC tissues by using the Kaplan–Meier curves (https://kmplot.com/analysis/, accessed on 1 July 2021).

### 4.10. Quantitative Reverse Transcription PCR (qRT-PCR)

The RNA extraction and qRT-PCR method were performed on previous experiments [12]. The primers used for qRT-PCR were as follows: MMP1: forward primer, 5′-CTT GCT CAT GCT TTT CGA CC-3′; reverse primer; 5′-TCC GGG TAG AAG GGA TTT GTG-3′; Glyceraldehyde 3-phosphate dehydrogenase (GAPDH): forward primer, 5′-CAT CAT CCC TGC CTC TAC TG-3′; and reverse primer, 5′-GCC TGC TTC ACC ACC TTC-3′ (MISSION BIOTECH, Taipei, Taiwan).

### 4.11. Statistical Analysis

Each experiment was repeated at least three times. The results are presented as the mean ± standard error. One-way analysis of variance followed by Dunnett post hoc test and statistical comparisons were conducted using Student’s *t*-test and SPSS (v.18.0). Significance was defined at the *p* < 0.05 or 0.01 levels.

## 5. Conclusions

In this study, CA was first reported to significantly inhibit cell migration and invasion of human OSCC cells in vitro. Analyses from the molecular mechanism revealed that CA suppressed the metastasis of OSCC cells through inhibition of the ERK1/2–MMP1 axis.

## Data Availability

Not applicable.

## Supplementary Materials

The following are available online at https://www.mdpi.com/article/10.3390/ijms22168641/s1, Figure S1: DMSO (0.04%) in culture medium did not affect the cell viability of HSC3 and SAS cells. Figure S2: Effect of scramble siRNA, siMMP1 and siERK2 on MMP1/ERK2 protein expressions and cell migration/invasion in HSC3 cells
