# Supplementary material for: Modulating the ERK1/2–MMP1 Axis through Corosolic Acid Inhibits Metastasis of Human Oral Squamous Cell Carcinoma Cells
Source: Int J Mol Sci. 2021 Aug 11;22(16):8641. doi: 10.3390/ijms22168641 (PMC8395509; doi:10.3390/ijms22168641)
Supplement: Supplementary file 1 [file ijms-22-08641-s001.zip › ijms-1304640-supplementary.pdf]

Modulating the ERK1/2–MMP1 Axis through Corosolic Acid Inhibits Metastasis of Human Oral Squamous Cell Carcinoma Cells

Jen-Liang Chen, Chung-Yu Lai, Tsung-Ho Ying, Chiao-Wen Lin, Pei-Han Wang, Fang-Jung Yu, Chung-Jung Liu, Yi-Hsien Hsieh

Supplementary Figure S1

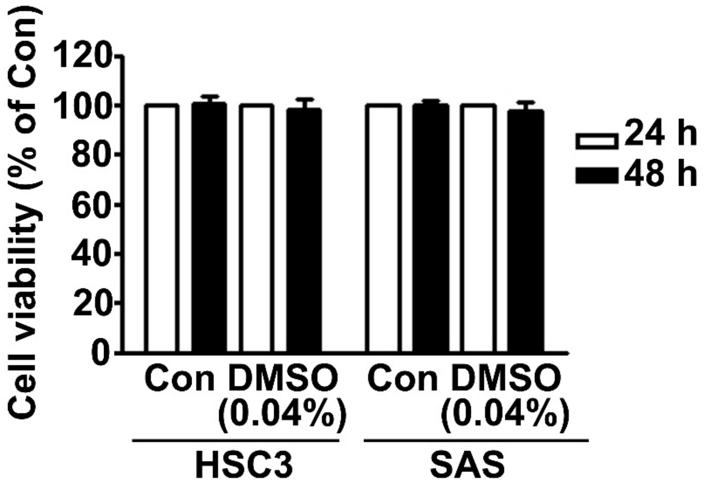

Supplementary Figure S1: DMSO (0.04%) in culture medium did not affect the cell viability of HSC3 and SAS cells. HSC3 and SAS cells were treated without DMSO (Con) or with DMSO (0.04%) for 24 and 48 h, cell viability was measured using the MTT assay.

Supplementary Figure S2

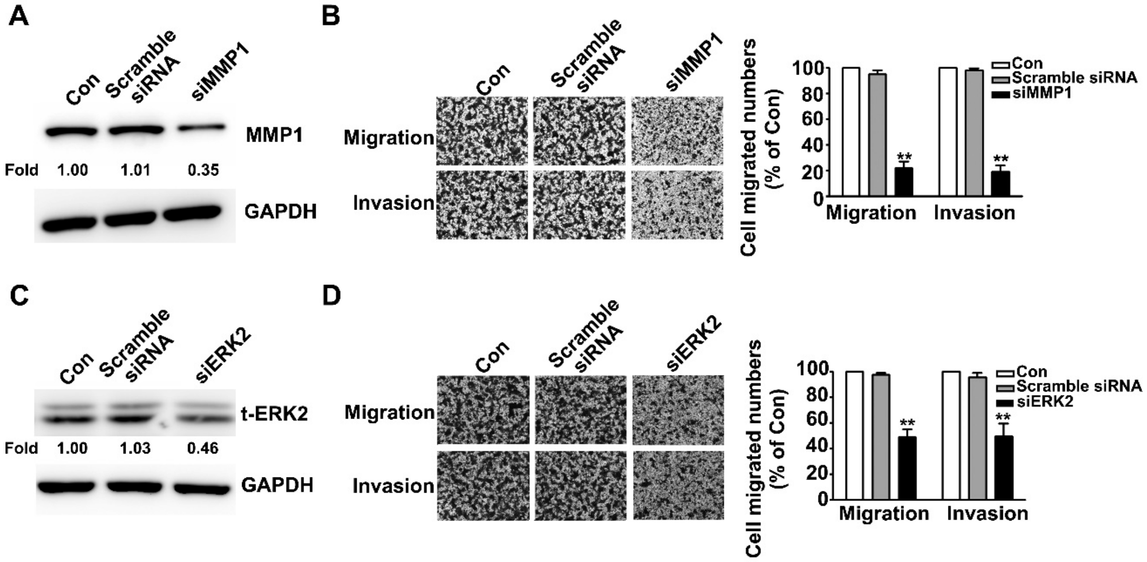

Supplementary Figure S2. (**A**, **C**) Transfection with scramble siRNA (100 nM), siMMP1(100 nM) or siERK2 (50 nM) into human HSC3 cells for 48 h. The MMP1/ERK2 protein expression by western blotting. (**B**, **D**) The cell migrated and invasive abilities of HSC3 cells were measured by in vitro migration and invasion assay. GAPDH as a protein-loading control. **, $p < 0.01$ versus con (untreated cells)
